# Silencing of prolyl endopeptidase protects against bone loss and enhances regeneration via bone anabolic and anti-catabolic effects

**DOI:** 10.1038/s41420-025-02905-y

**Published:** 2025-12-27

**Authors:** Huo-Liang Zheng, Hao Cai, Peng-Bo Chen, Lei-Sheng Jiang, Xin-Feng Zheng, Sheng-Dan Jiang

**Affiliations:** Department of Clinic of Spine Center, Xinhua Hospital, Shanghai Jiaotong University School of Medicine, Shanghai, 200082 China

**Keywords:** Cell biology, Physiology

## Abstract

Osteoporosis, characterized by diminished bone density and compromised microstructure, presents a significant healthcare challenge, particularly in the aging population. The primary approach in addressing osteoporosis involves the use of anti-resorptive agents as well as medications that promote bone formation. However, these therapies have limitations, prompting the exploration of novel therapeutic targets. Prolyl Endopeptidase (PREP), an endopeptidase with diverse roles in neuronal peptide metabolism and various physiological processes, has emerged as a potential player in osteoporosis, though its mechanistic involvement remains largely uncharted. This study delves into the role of PREP in osteoporosis, aiming to unravel its underlying mechanisms and therapeutic potential. Utilizing murine models and cellular experiments, we systematically investigate how PREP influences osteoblast differentiation and osteoporosis pathogenesis. Our results suggest activation of the Wnt pathway counteracts the inhibitory effects of PREP deletion on osteoblast differentiation. Additionally, we observe that PREP affects osteoclastogenesis, influencing osteoclast differentiation and bone resorption capacity. Moreover, our investigation extends to age-related osteoporosis, demonstrating PREP’s potential therapeutic efficacy beyond estrogen-deficiency-induced osteoporosis. In summary, this study advances our understanding of PREP’s multifaceted role in osteoporosis pathogenesis. It underscores PREP as a potential therapeutic target for osteoporosis, offering fresh perspectives on its etiology and treatment.

## Introduction

Osteoporosis, a pervasive skeletal disorder characterized by diminished bone density and compromised bone microstructure, substantially heightens the risk of fractures, particularly among middle-aged and elderly populations [1–4]. As the global demographic shifts towards an aging society, osteoporosis has emerged as a pressing public health concern, imposing substantial socioeconomic burdens [5, 6]. Consequently, in-depth investigations into osteoporosis pathophysiology and the development of innovative therapeutic strategies are of paramount importance.

The predominant therapeutic focus in managing osteoporosis centers on enhancing bone formation and inhibiting bone resorption to ameliorate bone density [7, 8]. Current treatment modalities primarily encompass anti-resorptive agents (such as bisphosphonates and estrogen replacement therapy) and bone-forming agents (such as teriparatide and abaloparatide) [9–13]. While these treatments have demonstrated efficacy in slowing osteoporosis progression, they are not without limitations, including adverse drug reactions and variable treatment outcomes. Consequently, the pursuit of novel treatment targets and strategies is essential to advance osteoporosis management.

Prolyl Endopeptidase (PREP), an endopeptidase responsible for cleaving peptide bonds adjacent to proline residues within polypeptide chains, plays a central biological role within the nervous system [14]. As a biomacromolecule, its involvement spans the metabolism and regulation of various neuropeptides and hormones, impacting critical physiological processes, including learning, memory, social behavior, emotional responses, stress modulation [15–17]. Moreover, PREP has been implicated in neurodegenerative disorders like Parkinson’s disease and autoimmune conditions like multiple sclerosis [18, 19]. Collagen serves as a crucial building block of the skeletal system, and it appears that PREP can also play a role in collagen hydrolysis [20, 21]. This association underscores the potential relevance of PREP in the context of osteoporosis.

This study hypothesizes that PREP, beyond its recognized neurophysiological roles, plays a crucial role in bone metabolism, impacting osteoblast differentiation and contributing to the pathogenesis of osteoporosis. Our primary aim is to elucidate the mechanistic role of PREP in osteoporosis through rigorous investigations utilizing murine models and cell-based experiments. Moreover, our study extends its reach to analyze the implications of PREP in the context of osteoporosis associated with aging. In addition to its recognized efficacy against estrogen-deficiency–induced osteoporosis, our data demonstrate that PREP deletion confers beneficial effects on age-related osteoporosis. This effect is characterized by increased bone mineral density, improved bone quality, and enhanced bone formation.

Our research broadens the understanding of PREP’s functionality, uncovering its pivotal role in osteoporosis pathogenesis and therapeutic potential. Our findings emphasize the significance of PREP in skeletal health and offer novel perspectives on osteoporosis etiology and treatment approaches. While our study primarily employs murine models, further research is imperative to validate the applicability of these findings in human populations. This study not only expands our knowledge of PREP’s multifaceted role but also highlights its potential as a therapeutic target for osteoporosis, thus paving the way for future investigations and clinical translation.

## Results

### PREP is downregulated during osteogenic differentiation

**Fig. 1:**
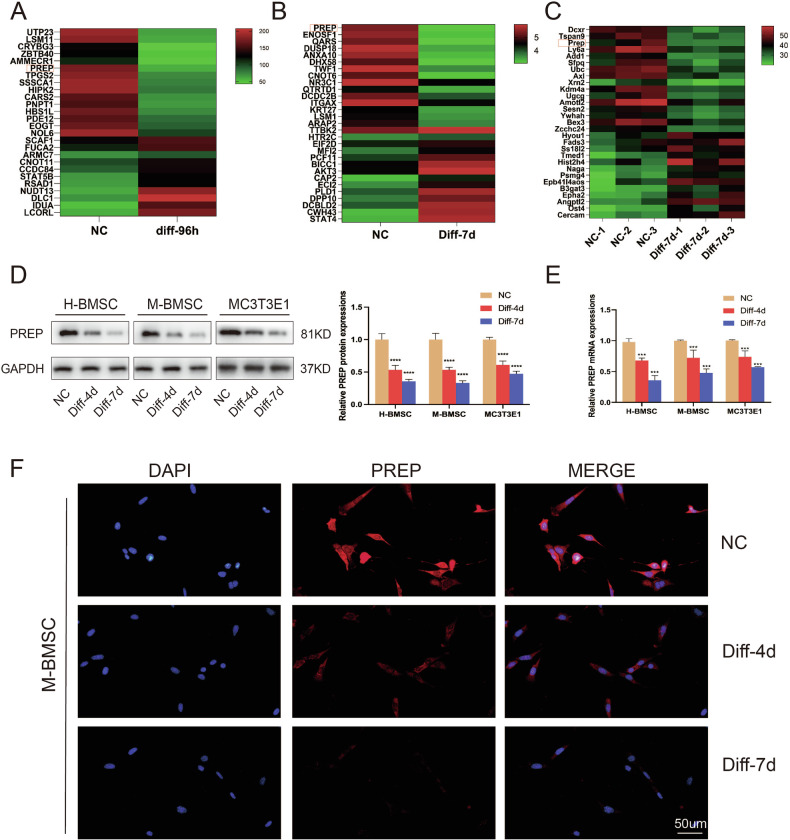
PREP is downregulated during osteogenic differentiation. **A**–**C** GEO database (GSE33567, GSE57265, and GSE154991) analysis indicates decreased expression of PREP compared to negative control (NC) during osteogenic differentiation. **D** Western blotting reveals decreased protein expression levels of PREP during osteogenic differentiation in human mesenchymal stem cells, mouse mesenchymal stem cells, and MC3T3E1 cells. **E** PCR reveals reduced mRNA expression of PREP during osteogenic differentiation in human mesenchymal stem cells, mouse mesenchymal stem cells, and MC3T3E1 cells. **F** Immunofluorescence confirms changes in PREP expression and shows PREP protein localization during osteogenic differentiation in mouse mesenchymal stem cells. Scale bars= 50 μm. *n* = 5, *P < 0.05, **P < 0.01 and ***P < 0.001 by *t* test.

To investigate the function of PREP in promoting osteogenic differentiation, a total of 3 sequencing data sets were analyzed in this study. Specifically, differential gene expression analysis (GSE33567) revealed PREP expression was decreased C2C12 cells treated with BMP-2 for 4 days (Fig. 1A). As shown in Fig. 1B, PREP expression was also down in h-BMSC 7 days after osteogenic differentiation (GSE57265). Again, the decreased expression of PREP was confirmed in primary mouse osteoblasts at day 7 of differentiation (Fig. 1C, GSE154991).

Following this lead, we next performed western-blotting and RT-qPCR assays to validate this decline. During the osteogenic differentiation of h-BMSC, m-BMSC, and MC3T3-E1 cells, PREP expression is reduced in not only mRNA level, but also in protein level (Fig. 1D, E). Additionally, this downregulation persisted throughout the whole differentiation epoch and showed a less dramatic decrease with differentiation progresses (Fig. 1F).

### PREP knockout prevents bone loss caused by ovariectomy

We initially examined the impact of PREP on osteoporosis induced by estrogen deficiency in a group of young females C57 mice (10 weeks old) undergoing ovariectomy (OVX), including both wild-type (WT) and PREP knockout mice. At 3 months post‑surgery, we measured uterine weight to evaluate the success of the surgery.

**Fig. 2:**
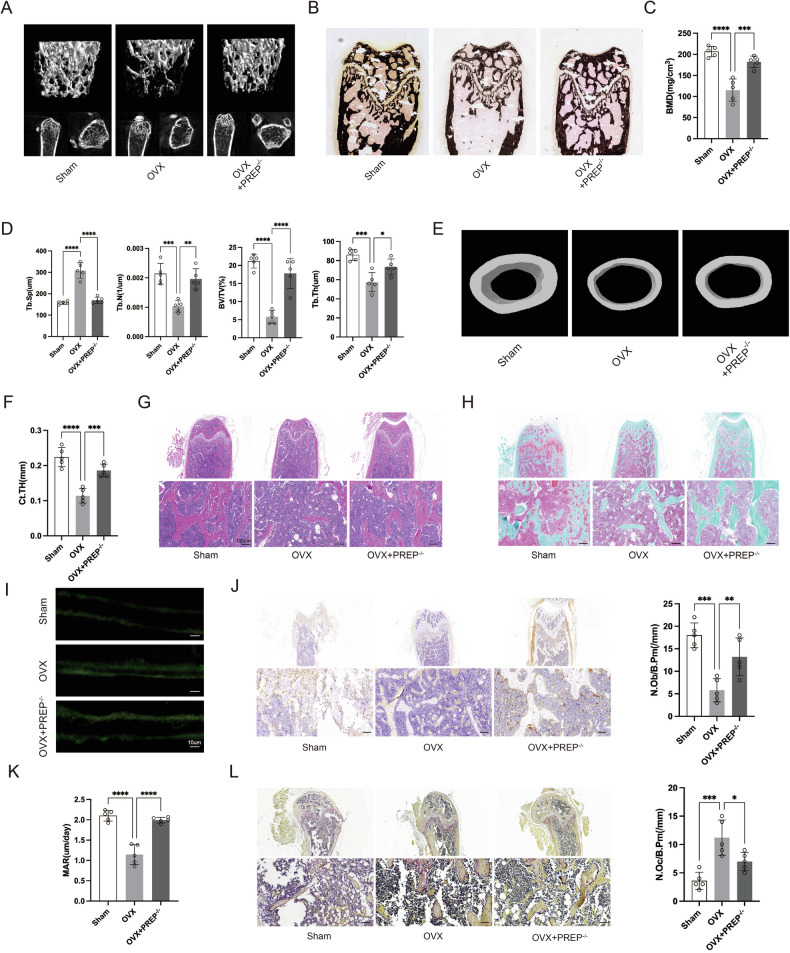
PREP knockout prevents bone loss induced by ovariectomy. **A** Three-dimensional reconstruction of the distal femur by micro-CT. **B** Silver-stained images of the distal femur in sham-operated, wild-type and RREP knockout osteoporotic model mice. **C**, **D** Comparison of BMD, Tb.Sp, Tb.N, BV/TV, and Tb.Th between sham-operated, wild-type and RREP knockout osteoporotic model mice. **E**, **F** Reconstruction images of cortical bone and comparison of cortical bone thickness between sham-operated, wild-type and RREP knockout osteoporotic model mice. **G**, **H** HE and Safranin O/Fast Green staining of the femur in sham-operated, wild-type and RREP knockout osteoporotic model mice. Scale bars = 100 μm. **I** Double-labeling with calcein and alizarin red in sham-operated, wild-type and RREP knockout osteoporotic model mice. Scale bars = 10 μm. **J** Immunohistochemical staining of Runx2 and quantification of osteoblast numbers in the distal femur of sham-operated, wild-type, and RREP knockout osteoporotic model mice. Scale bars = 100 μm. **K** Comparison of MAR between sham-operated, wild-type, and RREP knockout osteoporotic model mice. **L** TRAP staining and quantification of osteoclast numbers in the distal femur of sham-operated, wild-type, and RREP knockout osteoporotic model mice. Scale bars = 100 μm.

Our investigation, which involved von Kossa staining and micro-CT analysis of the distal femur metaphysis, found that compared with the sham-operated group, the OVX group showed significant decreases in bone mineral density (BMD), trabecular number (Tb.N), trabecular bone volume per tissue volume (BV/TV), and trabecular thickness (Tb.Th), whereas PREP knockout reversed these changes (Fig. 2A–D). A further observation was that PREP knockout markedly ameliorated the OVX-induced reduction in cortical thickness (Ct.TH) (Fig. 2E, F). HE staining and safranin O–fast green staining further confirmed the increases in cortical thickness and trabecular bone volume fraction in the PREP knockout group relative to the WT OVX group (Fig. 2G, H). Calcein double-labeling revealed that bone formation and mineral apposition rate were reduced in the WT OVX group, while both indices were significantly increased in the PREP knockout group compared with the WT OVX group (Fig. 2I, K). To further characterize osteogenic activity in PREP knockout mice, we performed immunohistochemistry to assess the expression of Runx2 (Runt-related Transcription Factor 2) in vivo. The results showed elevated Runx2 expression in PREP knockout mice relative to the WT OVX group, accompanied by increased osteoblast counts and reduced osteoclastic activity, as evidenced by tartrate‑resistant acid phosphatase (TRAP) staining (Fig. 2J, L).

### Deficiencies of PREP promote bone regeneration in vivo

**Fig. 3:**
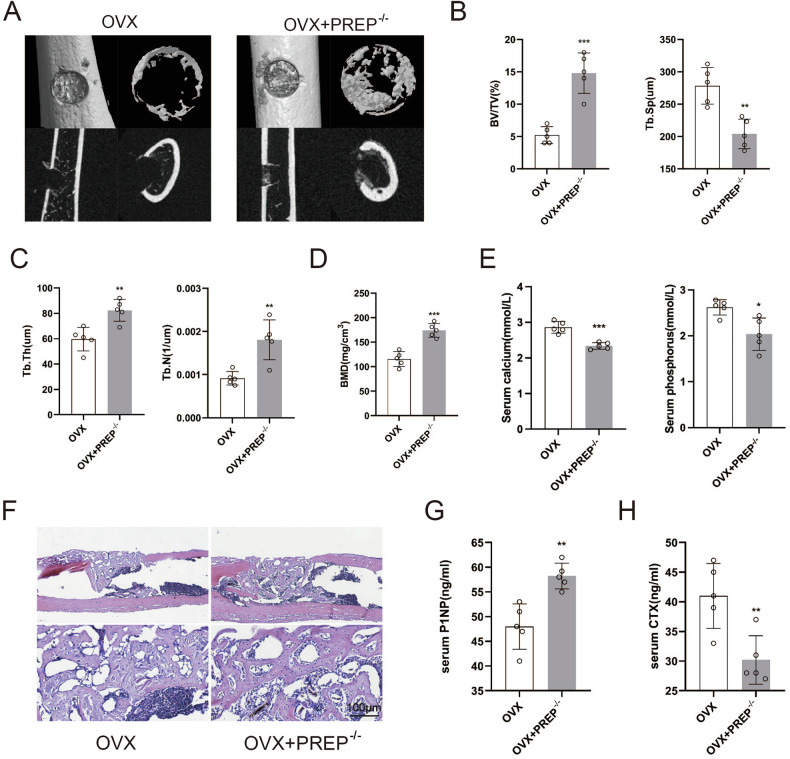
Deficiencies of PREP promote bone regeneration in vivo. **A** Three-dimensional reconstruction of femoral defects in wild-type and PREP knockout mice using micro-CT. **B**, **C** Measurements of Tb.Sp, Tb.N, BV/TV, and Tb.Th in femoral defects of wild-type and PREP knockout mice. **D** Bone density at the site of femoral defects in wild-type and PREP knockout mice. **E** Serum calcium and phosphorus in wild-type and PREP knockout mice. **F** Histological examination of femoral defects in wild-type and PREP knockout mice using HE staining. Scale bars = 100 μm. **G**, **H** Serum markers of bone resorption and bone formation in wild-type and PREP knockout mice. *n* = 5, *P < 0.05, **P < 0.01 and ***P < 0.001 by *t* test.

Following that, a mice cortical bone defect model was established to investigate the function of PREP in bone regeneration. Four weeks after ovariectomy, WT and PREP knocked out mice underwent the femoral cortical bone defect surgeries. One week following the operation, the femurs were harvested and examined. The results of the bone defects study matched our previous findings, micro-CT revealed that the cortical gaps in the PREP knockout group were nearly entirely bridged (Fig. 3A). As compared with the WT group, the PREP knockout group exhibited notably higher values for BMD, BV/TV, Tb. Th, and Tb. N in the mineralized callus (Fig. 3B–D). Simultaneously, there was a remarkable reduction in Tb.Sp in the PREP knockout group (Fig. 3B). We also observed that serum calcium and phosphorus levels were lower in PREP knockout mice than in the WT group (Fig. 3E). HE staining showed the bridge of fibered bone tissue extended across the entire cortical defect, again demonstrating that the deficiencies of PREP promote bone regeneration in vivo (Fig. 3F). As previously observed, PREP affected the osteogenic and osteoclastic activity. To probe this further, we employed Enzyme-Linked Immunosorbent Assay (ELISA) to determine the levels of cytokines associated with bone formation and resorption in serum. The serum Amino-terminal Propeptide of Type I Procollagen (P1NP) level was higher in PREP knockout group while C-terminal Telopeptides of Type I Collagen (β-CTX) level was declined (Fig. 3G, H).

### Osteoblastogenesis of BMSCs is inhibited in vitro by PREP

**Fig. 4:**
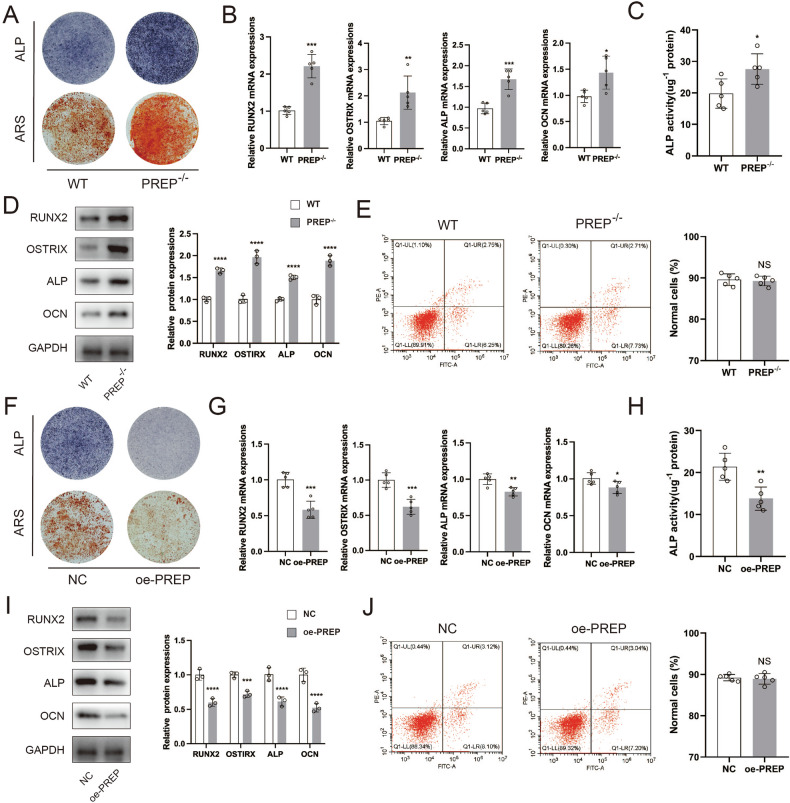
Osteoblastogenesis of BMSCs is inhibited in vitro by PREP. **A** Alizarin Red and ALP staining of BMSCs from wild-type and PREP knockout mice after osteogenic differentiation. **B** PCR analysis of the expression of osteogenic differentiation marker genes RUNX2, OSTERIX, OCN, and ALP in BMSCs from wild-type and PREP knockout mice. **C** Measurement of ALP activity in BMSCs from wild-type and PREP knockout mice. **D** Western blot analysis of the protein expression changes in osteogenic differentiation-related genes RUNX2, OSTERIX, OCN, and ALP in BMSCs from wild-type and PREP knockout mice. **E** Flow cytometry shows that PREP knockout does not affect apoptosis in BMSCs. **F** Alizarin Red and ALP staining of BMSCs with normal and overexpressed PREP after osteogenic differentiation. **G** PCR analysis of the expression of osteogenic differentiation marker genes RUNX2, OSTERIX, OCN, and ALP in BMSCs with normal and overexpressed PREP. **H** Measurement of ALP activity in BMSCs with normal and overexpressed PREP. **I** Western blot analysis of the protein expression changes in osteogenic differentiation-related genes RUNX2, OSTERIX, OCN, and ALP in BMSCs with normal and overexpressed PREP. **J** Flow cytometry shows that overexpression of PREP does not affect apoptosis in BMSCs. *n* = 5, *P < 0.05, **P < 0.01 and ***P < 0.001 by *t* test.

Subsequent to this, we undertook an examination of the effect of PREP knockout on the osteogenic differentiation of BMSCs. Both wild-type and PREP gene knockout mice were utilized to isolate bone marrow mesenchymal stem cells (BMSCs), which were subsequently subjected to osteogenic induction. Following this, assessment of osteogenic differentiation was conducted using Alizarin Red S (ARS) staining and Alkaline Phosphatase (ALP) staining. It was observed that osteogenic differentiation was significantly enhanced upon PREP knockout (Fig. 4A). During osteogenic differentiation, BMSCs derived from PREP knockout mice showed a marked increase in the expression of osteogenic differentiation markers, including RUNX2, OSTERIX, OCN (Osteocalcin), and ALP, when compared to their wild-type counterparts (Fig. 4B). This increase corresponded to a substantial elevation in ALP activity (Fig. 4C). Further Western-blotting assays demonstrated a concomitant rise in the protein expression levels of RUNX2, OSTERIX, OCN, and ALP after PREP knockout (Fig. 4D). To investigate the impact of PREP on apoptosis in BMSCs, we performed flow cytometric analysis, which showed that PREP knockout had no discernible effect on apoptosis in these cells (Fig. 4E).

In contrast, the osteogenic differentiation of BMSCs was hindered when PREP was overexpressed. Upon PREP overexpression, the expression of RUNX2, OSTERIX, OCN, and ALP was markedly diminished at both the RNA and protein levels (Fig. 4F–I). However, it was observed that the overexpression of PREP did not exert an impact on apoptosis in BMSCs (Fig. 4J).

### Osteogenic differentiation is modulated by PREP through engagement with the β-catenin signaling cascade

**Fig. 5:**
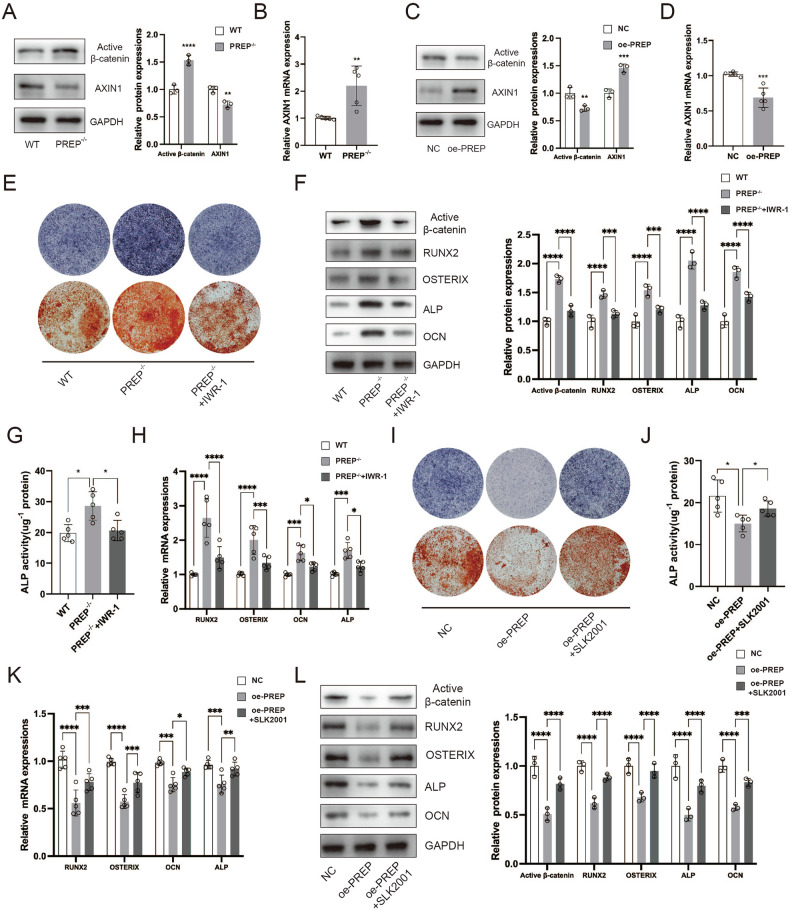
Osteogenic differentiation is modulated by PREP through engagement with the β-catenin signaling cascade. **A** Western blotting to assess changes in protein expression of Wnt pathway-related genes after PREP knockout. **B** PCR to examine changes in mRNA expression of Wnt pathway-related genes after PREP knockout. **C** Western blotting to evaluate changes in protein expression of Wnt pathway-related genes after PREP overexpression. **D** PCR to investigate changes in mRNA expression of Wnt pathway-related genes after PREP overexpression. **E** ALP and ARS staining to assess the impact of IWR-1 on osteogenic differentiation in PREP knockout BMSCs. **F** Western blotting to investigate the impact of IWR-1 on the expression of osteogenic differentiation-related proteins and the Wnt pathway in PREP knockout BMSCs. **G** Measurement of ALP activity to determine the influence of IWR-1 on ALP activity in PREP knockout BMSCs. **H** PCR analysis of the effect of IWR-1 on the expression of osteogenic differentiation-related genes (RUNX2, OSTERIX, OCN, and ALP) in PREP knockout BMSCs. **I** ALP and ARS staining to assess the effect of SLK2001 on osteogenic differentiation in BMSCs with overexpressed PREP. **J** Measurement of ALP activity to determine the influence of SLK2001 on ALP activity in BMSCs with overexpressed PREP. **K** PCR analysis of the effect of SLK2001 on the expression of osteogenic differentiation-related genes (RUNX2, OSTERIX, OCN, and ALP) in BMSCs with overexpressed PREP. **L** Western blotting to investigate the impact of SLK2001 on the expression of osteogenic differentiation-related proteins and the Wnt pathway in BMSCs with overexpressed PREP. *n* = 5, *P < 0.05, **P < 0.01, and ***P < 0.001 by *t* test.

We evaluated the influence of PREP on osteogenesis-related pathways and observed a substantial rise in the expression of activated β-catenin following PREP knockout. The β-catenin pathway assumes a central role in the regulation of osteogenic differentiation and the formation of bone tissue. Consequently, we hypothesized that PREP potentially modulates osteogenic differentiation through the β-catenin signaling pathway. Following PREP knockout, there was a notable reduction in the expression of AXIN1, an upstream regulatory gene of β-catenin, subsequently resulting in elevated expression of activated β-catenin (Fig. 5A, B). Conversely, overexpressing PREP led to a promotion in AXIN1 RNA expression, correspondingly causing a decrease in activated β-catenin expression (Fig. 5C, D). To further substantiate our hypothesis, we introduced the β-catenin pathway inhibitor, IWR-1, during osteogenic differentiation. As depicted in Fig. 5E, the enhanced osteogenic activity resulting from PREP knockout was attenuated upon IWR-1 administration. Moreover, the expression of RUNX2, OSTERIX, OCN, and ALP notably diminished under IWR-1 stimulation (Fig. 5F–H). Conversely, when utilizing the β-catenin pathway agonist SLK2001, the reduced osteogenic activity attributed to PREP overexpression was reinvigorated (Fig. 5I–L). These discoveries emphasize the substantial contribution of the β-catenin signaling pathway to the modulation of osteogenic differentiation by PREP.

### Osteoclastogenesis process is affected by PREP

**Fig. 6:**
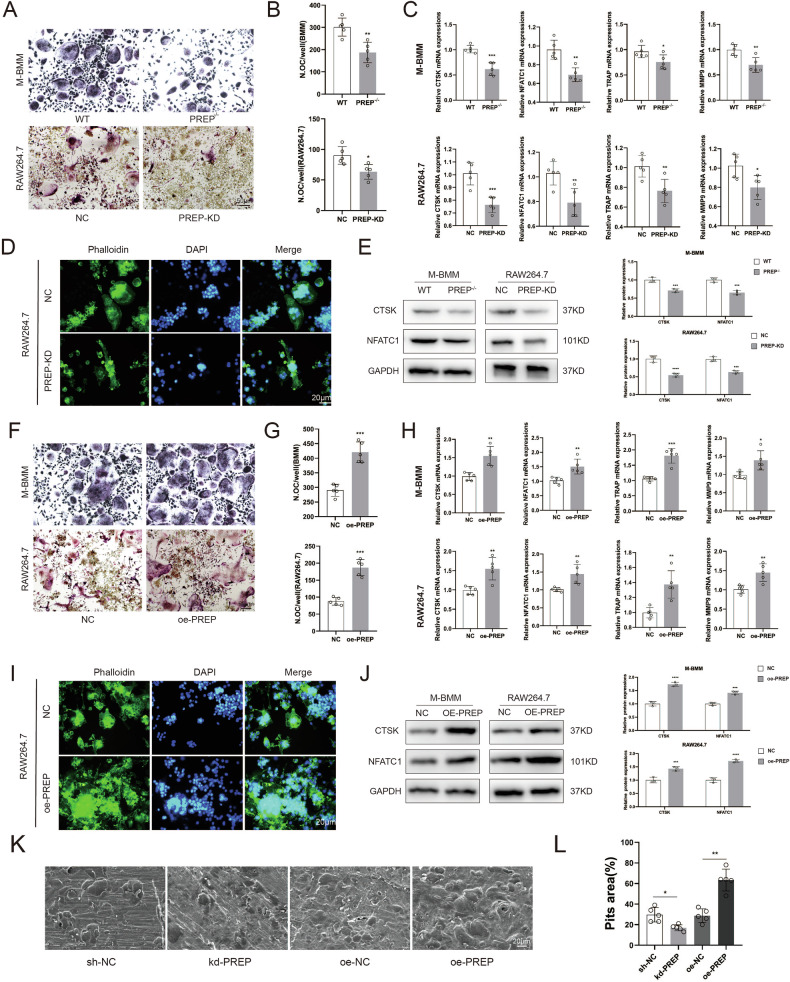
Osteoclastogenesis process is affected by PREP. **A** TRAP staining to assess the impact of PREP knockout or knockdown on osteoclast differentiation in mouse bone marrow mononuclear cells and raw264.7 cell line. Scale bars= 50 μm. **B** Quantification of the number of osteoclasts. **C** PCR analysis of the mRNA expression of osteoclast differentiation-related genes in mouse bone marrow mononuclear cells and raw264.7 cell line after PREP knockout or knockdown. **D** Fluorescent staining with phalloidin to show that PREP knockdown inhibits cell fusion in raw264.7 cells. Scale bars = 20 μm. **E** Western blotting to investigate osteoclast differentiation-related proteins in mouse bone marrow mononuclear cells and raw264.7 cell line after PREP knockout or knockdown. **F** TRAP staining to assess the impact of PREP overexpression on osteoclast differentiation in mouse bone marrow mononuclear cells and raw264.7 cell line. Scale bars = 50 μm. **G** Quantification of the number of osteoclasts formed with PREP overexpression. **H** PCR analysis of the mRNA expression of osteoclast differentiation-related genes in mouse bone marrow mononuclear cells and raw264.7 cell line after PREP overexpression. **I** Fluorescent staining with phalloidin to show that PREP overexpression promotes cell fusion in raw264.7 cells. Scale bars = 20 μm. **J** Western blotting to investigate osteoclast differentiation-related proteins in mouse bone marrow mononuclear cells and raw264.7 cell line after PREP overexpression. **K**, **L** Bone resorption assays indicating that PREP enhances osteoclasts’ bone resorptive capability. Scale bars = 20 μm. *n* = 5, *P < 0.05, **P < 0.01 and ***P < 0.001 by *t* test.

Initially, we downregulated the expression of PREP in the RAW264.7 cells and induced osteoclast differentiation. Additionally, we employed bone marrow monocytes from PREP knockout mice as primary cells to induce osteoclastogenesis. Evidently, reduced PREP expression significantly suppressed the osteoclast differentiation capability in both RAW264.7 cells and monocytes (Fig. 6A, B). Subsequent PCR experiments further revealed that the downregulation of PREP led to a marked inhibition in the expression of osteoclast differentiation marker genes, including Cathepsin K (CTSK), Nuclear Factor of Activated T Cells, Cytoplasmic 1 (NFATc1), TRAP, and Matrix Metalloproteinase 9 (MMP9) (Fig. 6C). Fluorescent staining indicated that PREP knockdown resulted in a reduced volume of osteoclasts differentiated from RAW264.7 cells (Fig. 6D). Western blot results further demonstrated that PREP knockout inhibited osteoclast differentiation (Fig. 6E). Conversely, upon overexpressing PREP in both RAW264.7 cells and monocytes, their osteoclast differentiation capacity was significantly enhanced (Fig. 6F, G). The expression of osteoclast differentiation marker genes (CTSK, NFATc1, TRAP, and MMP9) increased concomitantly with elevated levels of PREP expression (Fig. 6H). Fluorescent staining indicated that heightened PREP expression led to an enlarged volume of osteoclasts differentiated from RAW264.7 cells (Fig. 6I). Western blot results showed that PREP overexpression promoted the expression of proteins involved in osteoclast differentiation (Fig. 6J).

Following that, we assessed the impact of PREP on osteoclast functionality. As shown in Fig. 6K, osteoclasts overexpressing PREP demonstrated an increased bone resorption capacity, resulting in larger resorption pit areas on bone slices. In contrast, upon PREP knockdown, the area of bone resorption pits showed a significant reduction (Fig. 6L). In summary, these findings highlight the pivotal role of PREP in regulating osteoclast differentiation and functional attributes, influencing both their differentiation propensity and bone resorption capacity.

### PREP knockout alleviates age-related osteoporosis

**Fig. 7:**
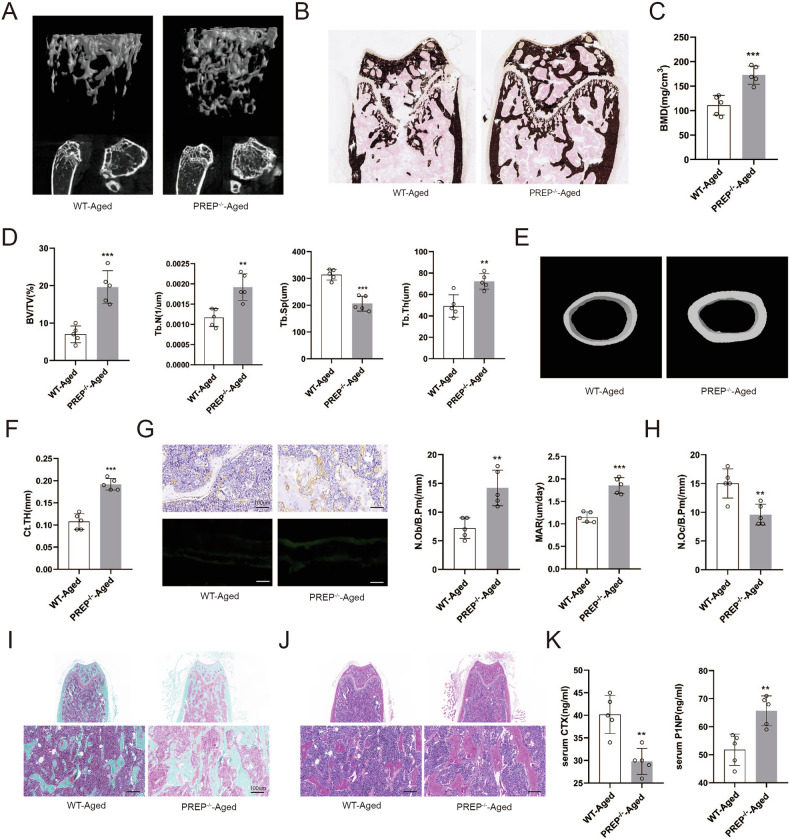
PREP knockout alleviates age-related osteoporosis. **A** Three-dimensional reconstruction of the femur by micro-CT in aged wild-type and PREP knockout mice. **B** Silver staining images of the distal femur in aged wild-type and PREP knockout mice. **C** Measurement of BMD in aged wild-type and PREP knockout mice. **D** Comparison of Tb.Sp, Tb.N, BV/TV, and Tb.Th between aged wild-type and PREP knockout mice. **E**, **F** Reconstruction images of cortical bone and comparison of cortical bone thickness in aged wild-type and PREP knockout mice. **G** Immunohistochemical staining of Runx2, double labeling with calcein and alizarin red, quantification of osteoblast numbers, and mineralization rate in aged wild-type and PREP knockout mice. Scale bars = 100 μm. **H** Quantification of osteoclast numbers in aged wild-type and PREP knockout mice. **I**, **J** HE and Safranin O/Fast Green staining of the femur in aged wild-type and PREP knockout mice. Scale bars = 100 μm. **K** Serum markers of bone resorption and formation in aged wild-type and PREP knockout mice. *n* = 5, *P < 0.05, **P < 0.01, and ***P < 0.001 by *t* test.

Subsequently, we investigated the impact of PREP on age-related osteoporosis. As depicted in Fig. 7A, PREP knockout effectively ameliorated age-related osteoporosis. Von Kossa assays further indicated enhanced femur bone mass in PREP knockout mice (Fig. 7B). Bone density, trabecular number, trabecular volume fraction, and trabecular thickness were all elevated in PREP knockout mice compared to the wild-type group (Fig. 7C, D). Additionally, cortical bone thickness was increased in PREP knockout mice (Fig. 7E, F). In terms of bone formation rate, PREP knockout mice also outperformed the wild-type mice (Fig. 7G). Immunohistochemical analysis post PREP knockout demonstrated a significant increase in Runx2 expression (Fig. 7G). This suggests a potential regulatory role of PREP in osteoblast and osteoclast activity in aged mice in vivo (Fig. 7G, H). Further corroborating evidence was derived from both HE staining and SafraninO-Fast Green staining, confirming a noteworthy elevation in trabecular thickness and number in aged mice upon PREP knockout (Fig. 7I, J).

Serum samples extracted from aged wild-type mice and PREP mice were employed for assessing bone metabolism markers. ELISA assay results demonstrated a significant reduction in β-CTX and a substantial elevation in P1NP in aged PREP knockout mice (Fig. 7K).

These findings underscore the favorable influence of PREP knockout on age-related osteoporosis. Enhanced skeletal parameters, improved trabecular thickness and number, as well as altered serum bone metabolism markers collectively point towards the regulatory role of PREP in osteoblast and osteoclast activities in the context of aging.

## Discussion

Osteoporosis, a common skeletal disorder, is particularly prevalent among middle-aged and elderly women [22–24]. With advancing age, the incidence of osteoporosis steadily increases, resulting in an elevated risk of fractures and imposing a substantial socioeconomic burden [25, 26]. Osteoporosis is typically characterized by diminished bone density and compromised bone microarchitecture. Collagen, as a primary protein component, plays a pivotal role in bone tissue, constituting a vital fraction of the bone matrix [27, 28]. Furthermore, collagen assumes a crucial function during fracture healing and bone regeneration processes [29]. It provides structural support for nascent bone tissue, facilitating the reparative and regenerative phases. PREP, an endopeptidase capable of selectively cleaving carboxyl-terminal peptide bonds of peptides with prolyl residues, significantly contributes to collagen degradation. FUKUOKA’s research has unveiled a potential close association between PREP and murine arthritis [30]. The linkage between PREP and Parkinson’s disease has also been previously substantiated by Svarcbahs [18]. Additionally, Tommi P. Kilpeläinen and colleagues have provided evidence for a potential connection between PREP and autophagy [31]. Moreover, Reinis Svarcbahs claims PREP inhibition activates autophagy via protein phosphatase 2A [32]. However, the potential relationship between PREP and osteoporosis has not been comprehensively investigated in the current literature. The interplay among osteoporosis and the role of PREP remains an uncharted territory within the realm of scientific inquiry. Further research into this nexus holds substantial promise for elucidating novel mechanisms underlying osteoporosis and may pave the way for innovative therapeutic interventions in the future.

Our research has unveiled that the downregulation of PREP in osteogenic differentiation may represent a pivotal regulatory mechanism. Through an extensive analysis encompassing diverse cellular models and murine models, we have substantiated that the diminished expression of PREP correlates with augmented bone formation and reduced bone resorption. In particular, we have elucidated that PREP knockout mice exhibit remarkable bone protective effects post-ovariectomy surgery. This phenomenon can be elucidated through the augmentation of bone density, the enhancement of bone microarchitecture, and the promotion of bone formation. Additionally, our findings indicate that PREP knockout can also facilitate in vivo bone regeneration, manifested as accelerated bone defect healing and increased bone density. In terms of molecular mechanisms, osteoblast differentiation is regulated by various signaling pathways. For instance, the BMP signaling pathway, when activated, can promote the nuclear translocation of smads proteins, thereby regulating the process of osteoblast differentiation [33–35]. The Notch signaling pathway can influence osteoblast differentiation by impacting the transcription of target genes such as Hes and Hey [36, 37]. Many studies have suggested that the PI3K-AKT signaling pathway also plays a significant role in osteoblast differentiation [38–40]. Although previous research has demonstrated that PREP can affect the activity of the PI3K-AKT signaling pathway, our experiments suggest that PREP does not appear to influence osteoblast differentiation through the PI3K-AKT pathway [41]. Following PREP regulation, the expression levels of Akt remained unchanged. Our research suggests that PREP may modulate osteogenic differentiation through its influence on the β-catenin signaling pathway. The Wnt pathway is widely recognized as a crucial regulatory factor in bone formation, and our experimental results indicate that PREP knockout leads to the activation of β-catenin, thereby enhancing osteogenic differentiation. Furthermore, upon the administration of a Wnt pathway agonist, the inhibitory effect of PREP on osteoblast differentiation was counteracted. Conversely, when a Wnt pathway inhibitor was employed, the enhanced osteoblast differentiation resulting from PREP knockout was once again suppressed. This discovery provides a compelling clue for a deeper understanding of the mechanistic role of PREP in osteoporosis.

**Fig. 8:**
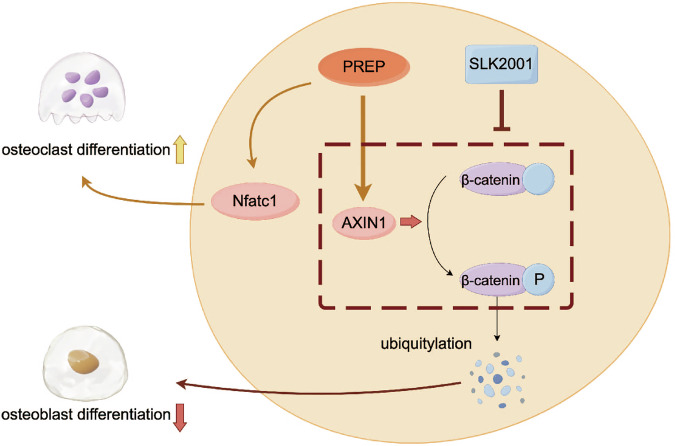
PREP induces osteoclast differentiation and inhibits osteoblast differentiation through AXIN1.

Over the last two decades, there has been a significant emphasis within the field of osteoclast differentiation on the role of RANKL induction. This includes a comprehensive investigation into the intricate signaling pathways involved, as well as a comprehensive exploration of key osteoclastogenic transcription factors. Notably, these factors encompass NFATc1, CTSK, and TRAP, representing a classic ensemble that has garnered substantial attention in osteoporosis. In this study, we have also observed the impact of PREP on osteoclasts. The knockdown of Prep expression has been shown to significantly inhibit the expression of key factors associated with osteoclast differentiation. Here, we do not rule out the possibility that PREP knockout could restrict collagen degradation, thereby impacting the bone resorption process. Our primary focus is directed towards investigating PREP as a bioactive factor that modulates signaling within cells and influences differentiation functions. As shown in our results, PREP knockout impedes osteoclast differentiation and function, diminishing bone resorptive capacity. This further underscores the significant role of PREP throughout the entire pathophysiological process of osteoporosis. In addition to its significant role in estrogen-deficiency-induced osteoporosis models, we further examined the impact of PREP on age-related osteoporosis. Our research results suggest a potential role for PREP in age-related osteoporosis. PREP knockout ameliorates bone density and structure in elderly mice, reduces bone resorption, and promotes bone formation. This implies that PREP may exert a regulatory effect in osteoporosis across different age groups, and also indicate that PREP knockout exhibits promising therapeutic effects in both types of osteoporosis. These findings collectively shed light on the multifaceted involvement of PREP in bone health and underscore its potential as a therapeutic target in the context of osteoporosis (Fig. 8).

While our paper mentions that the Wnt signaling pathway plays a major role in mediating the molecular mechanisms through which PREP primarily influences osteoblast differentiation, further in-depth research and elucidation are needed to precisely understand how PREP regulates the Wnt pathway. Additionally, the potential applications of PREP in osteoporosis and its clinical translation may require further investigation. Inhibiting PREP may also affect other normal physiological functions. Therefore, precise targeting of osteoclasts is a critical issue for the clinical translation of PREP. Its therapeutic efficacy should also be compared with existing agents, including bisphosphonates and RANKL monoclonal antibodies. Moreover, this study primarily used female mouse models. On the one hand, the role of PREP in male mice has not been further validated. On the other hand, we also need to acknowledge the physiological and pathological differences between adult mice and humans. In summary, our study provides a new direction and target for future anti-osteoporosis therapies, and more extensive research is warranted to validate the applicability of these findings in humans.

## Materials and methods

### Cell culturing

m-BMSCs, MC3T3-E1 cells (ATCC), and H-MSCs were cultured in Dulbecco’s modified Eagle’s medium (DMEM, #11995040, Thermo Fisher), supplemented with 10% fetal bovine serum (FBS, #10099141 C, Thermo Fisher, USA), 100 U/mL penicillin (Invitrogen, USA), and 100 μg/mL streptomycin (Invitrogen, USA). These cultures were maintained at 37 °C in a humidified environment with 5% CO2.Raw264.7 cells (ATCC) and bone mouse-marrow-derived macrophages (m-BMMs) were cultured in α-MEM (#12571063, Thermo Fisher), also supplemented with 10% FBS.

### Immunohistochemistry (IHC) and immunofluorescence (IF) staining

To prepare for IHC and IF staining, we first fixed the bone tissue sections in 10% buffered formalin and then proceeded to embed them in paraffin. Slides underwent deparaffinization in xylene and subsequent rehydration using alcohol for both IHC and IF staining procedures. In the context of IHC, we utilized a 3% hydrogen peroxide solution to inhibit endogenous peroxidase activity, and we performed antigen retrieval by subjecting the samples to microwave irradiation in a 0.1 M citric sodium buffer. Subsequently, the sections were treated with a 30-min incubation in 5% BSA, and then they were subjected to an overnight exposure to the primary antibody at 4 °C. Following three rinses with phosphate buffered saline (PBS), we employed an HRP-DAB kit (KIT-5920, MaxvisionTM2 HRP-Polymer anti-Mouse/Rabbit IHC Kit) to visualize antibody binding, and hematoxylin was used for counterstaining. In IF staining, we used a secondary antibody labeled with a fluorescent marker to bind to the primary antibody, and we conducted image acquisition using an Olympus microscope (Olympus BX51).

### Animals

Approval for all animal studies was obtained from the Ethics Committee of Xinhua Hospital, which is affiliated with the Shanghai Jiao Tong University School of Medicine. Female C57BL mice, with an age of 10 weeks and weighing between 20 and 25 grams, were obtained from Shanghai Jihui Laboratory Animal Care Co. Ltd. Accommodated in pathogen-free facilities, the mice followed a 12-h light and 12-h dark cycle. In the initial phase, 10 mice were randomly allocated to two groups (*n* = 5 per group) to investigate the effects of PREP on bone mass. Ovariectomy was performed on all of the mice. Afterward, an extra 10 female mice were randomly distributed among three groups (*n* = 5 per group) for the purpose of bone defect assays. In the study, elderly mice, aged 20 months, were employed as a model to investigate age-related osteoporosis.

### Calcein double labeling and Micro-CT analysis

The mice received calcein at a dosage of 10 mg/kg, with injections administered 8 and 3 days prior to their sacrifice. Following euthanasia, the femurs were promptly extracted and then immersed in 70% ethanol for a period of 24 h. Subsequently, they were subjected to scanning using a Scanco µCT 40 scanner at a resolution of 18 µm. We defined the region of interest (ROI) as the trabecular bone positioned 1 mm beneath the epiphyseal growth plate. The bone specimens underwent a sequential processing sequence, including steps with 90% ethanol, 100% ethanol, LR white hydrophilic medium, and a final heating step at 60 °C for 12 h. Subsequently, we sliced sections with a thickness of 6 µm utilizing a LEICA SP1600 Saw Microtome and subjected to imaging under fluorescence microscopy (Olympus BX51).

### ELISA

To quantitatively analyze P1NP, β-CTX, and RANKL, we employed Enzyme-Linked Immunosorbent Assay (ELISA) methodology. The concentrations of cytokines were assessed using ELISA kits sourced from ELISAGenie. All procedures were meticulously conducted in strict accordance with the manufacturer’s instructions. It is noteworthy that mice were subjected to a 6-h fasting period prior to blood collection.

### Osteogenic differentiation, ALP, and ARS staining

Female C57BL/6 mice, aged 4 weeks, were the source of primary m-BMSCs isolated from bone marrow. Following the retrieval of the cell suspension through bone marrow flushing, it was cultured in DMEM containing 10% FBS. Cells from the second passage were employed in our osteoblast differentiation experiments and seeded in 6-well plates at a density of 5000 cells/cm². In the osteogenic differentiation medium, comprising 10 mM β-glycerophosphate, 0.1 μM dexamethasone, and 0.05 mM ascorbic acid, the cells were cultured until they reached 80% confluence. Every two days, the culture medium was renewed. ALP and ARS staining were conducted on the 7th and 14th days of osteogenesis, respectively. To conduct ALP staining, we first fixed the cells using 4% paraformaldehyde and subsequently incubated them at 37 °C in 0.1 M Tris buffer (pH 9.3), which contained 0.25% naphthol AS-BI phosphate and 0.75% Fast Blue BB. In the case of ARS assays, the cells were fixed with 4% paraformaldehyde after two PBS washes and subsequently stained with 1% Alizarin Red S (pH 4.2, Sigma-Aldrich) for a duration of 10 min.

### Surgeries

Randomization was performed using a computer-generated random sequence. Anesthesia for the mice was induced using ketamine (120 mg/kg, Medistar) and xylazine (16 mg/kg, Riemser). For bilateral ovariectomy, the procedure entailed making a midline dorsal skin incision and conducting dissection of the muscle layer. Subsequently, the ovaries were ligated and excised. Before euthanizing the mice, the uteri were excised and subjected to weighing for gravimetric analysis. The uterine weight served as an indicator to assess the success of the ovariectomy (OVX) procedure. To create femoral cortical bone defects, we initiated the process by making a skin incision and gently dissecting the quadriceps. Subsequently, we exposed the surfaces of the mid-femurs. Using a round bur, we drilled a hole (0.9 mm diameter and 1 mm depth). The incision was then closed with silk sutures. Mice with postoperative wound infection after femoral drilling (trochanteric fenestration) or with complete femoral discontinuity/fracture were excluded from the analysis.

### Osteoclastogenic differentiation and TRAP staining

Female C57BL/6 mice, aged 4 weeks, served as the source for primary m-BMMs isolated from bone marrow. The cell suspension was cultured in α-MEM medium. To prompt the differentiation process leading to BMMs, the culture medium was augmented with 30 ng/mL of Macrophage Colony-Stimulating Factor (M-CSF), maintained for a 3-day period. Subsequently, cells, either Raw264.7 or m-BMMs derived from mouse femurs, were cultivated in 48-well plates, with each well containing 2 × 10^4^ cells. RAW264.7 cells were exposed to 100 ng/mL RANKL. The incubation process for m-BMMs included the presence of both 100 ng/mL RANKL and 30 ng/mL M-CSF. Seven days into the induction process, the cells were rinsed and then subjected to fixation with 4% paraformaldehyde for a half-hour interval. Following fixation, the cells were stained for TRAP using a commercially available kit (Sigma-Aldrich). Osteoclasts were identified as TRAP+ cells containing a minimum of three nuclei, and their quantification was performed using a microscope (Olympus BX51).

### Quantitative RT-PCR

RNA extraction from various cell types was carried out using TRIzol reagent (Invitrogen). Post-lysis using TRIzol, we introduced chloroform to the samples, and then they were centrifuged at 12,000 × *g* for 15 min. To the upper, transparent solution containing RNA, an equal volume of isopropanol was subsequently introduced. After another round of centrifugation, the RNA pellet was subjected to two washes with 75% ethanol. To facilitate reverse transcription, RNA was converted into cDNA using PrimeScript™ RT Master Mix (Takara, RR036A). Real-time PCR was conducted using HieffTM PCR Master Mix (Yeasen). Subsequently, the normalization of the target genes’ expression was carried out with reference to the expression of GAPDH.

### Western blotting

To achieve cell lysis, we utilized radioimmunoprecipitation assay (RIPA) buffer (Beyotime, P0013C) on ice, with the addition of PMSF (Beyotime, ST506). For protein separation within the lysates, we employed 10% sodium dodecyl sulfate polyacrylamide gel electrophoresis (SDS-PAGE). Afterward, we transferred these proteins onto PVDF membranes (0.22 μm, Millipore) and proceeded with a 15-min blocking process at room temperature, employing Free Quick Blocking Buffer (Epizyme, PS108P). Next, the membranes were subjected to an overnight incubation at 4 °C with primary antibodies, followed by a series of three washes with TBST. Afterward, at room temperature, the membranes were incubated with HRP-conjugated secondary antibodies (Beyotime) for a duration of 1 h. The identification of antibody-antigen complexes was accomplished through the use of an ECL reagent (Epizyme). Primary antibodies used in this study included those against Runx2 (#12556), TRAF6 (#8028), NFATc1 (#8032), GAPDH (#2118), all sourced from Cell Signaling Technology (Boston, United States). Additionally, antibodies targeting OSTERIX (#ab209484), ALP (#ab229126) and OCN (#ab133612) were procured from Abcam (Cambridge, United Kingdom). Antibody targeting PREP was purchased from Proteintech (11536-1-AP).

### Resorption pit assay

The resorption pit assay was employed to assess osteoclast function. BMMs were placed onto bovine bone slices in a 96-well plate, with each well containing 1 × 10^4^ cells. Moreover, for the monitoring of osteoclast morphology throughout the differentiation process, we incorporated wells without bovine bone slices but with BMMs. BMMs were exposed to 100 ng/mL RANKL and 30 ng/mL M-CSF for a 7-day period, following the previously mentioned procedure, to induce the formation of mature osteoclasts. Subsequently, the cells were softly brushed off the bone slices’ surface. Next, the bone slices were subjected to gold coating on their surface and photographed using a scanning electron microscope (FEI Instr. Software, Hillsboro, OR USA) to visualize and analyze the resorption pits within the slices.

### Transfection of lentivirus or siRNA

Overexpression of PREP (oe-PREP) was achieved through lentiviral transduction. Prior to lentivirus transfection, the cells were seeded at an initial density of 40% in six-well plates. Lentiviruses encoding PREP were introduced into BMSCs in serum-free DMEM medium, along with polybrene. The culture medium was switched to standard DMEM with 10% FBS one day after transduction. The selection process with puromycin (Beyotime) was commenced 48 h after transfection of the cells. The initial step involved plating cells in six-well plates at a density ranging from 30% to 50 for siRNA transfections. Following this, both Lipofectamine 2000 (Thermo Fisher Scientific) and siRNA were diluted with serum-free DMEM medium. After a 5-min incubation period, the siRNA and Lipofectamine 2000 dilutions were combined and allowed to sit at room temperature for 20 min. Following the preparation of the mixture, it was incorporated into cells for transfection. The day after, the culture medium was changed to standard DMEM with 10% FBS.

### Statistical analysis

Statistical analysis was carried out, with each experiment having a minimum of three independent replicates. We determined sample size based on a pre-specified effect size, two-sided testing, α = 0.05, and power 1−β = 0.8. Data are presented as mean ± standard deviation (SD). During the experiment and outcome assessment, investigators were blinded to group allocation to the following extent: operators were blinded during data acquisition, and statisticians remained blinded until the primary analysis was finalized. To evaluate statistical significance between two groups, a two-tailed Student’s *t* test was employed. For comparisons involving multiple groups, one-way analysis of variance (ANOVA) or two-way ANOVA was applied, followed by Tukey’s test. A level of statistical significance was determined with a threshold of *P* < 0.05.

## Supplementary information

original wb

## Data Availability

The datasets used and analyzed during the current study are available from the corresponding author on reasonable request.
