# Supplementary figures and images for: Silencing of prolyl endopeptidase protects against bone loss and enhances regeneration via bone anabolic and anti-catabolic effects
Source: Cell Death Discov. 2025 Dec 27;12:51. doi: 10.1038/s41420-025-02905-y (PMC12848018; doi:10.1038/s41420-025-02905-y)

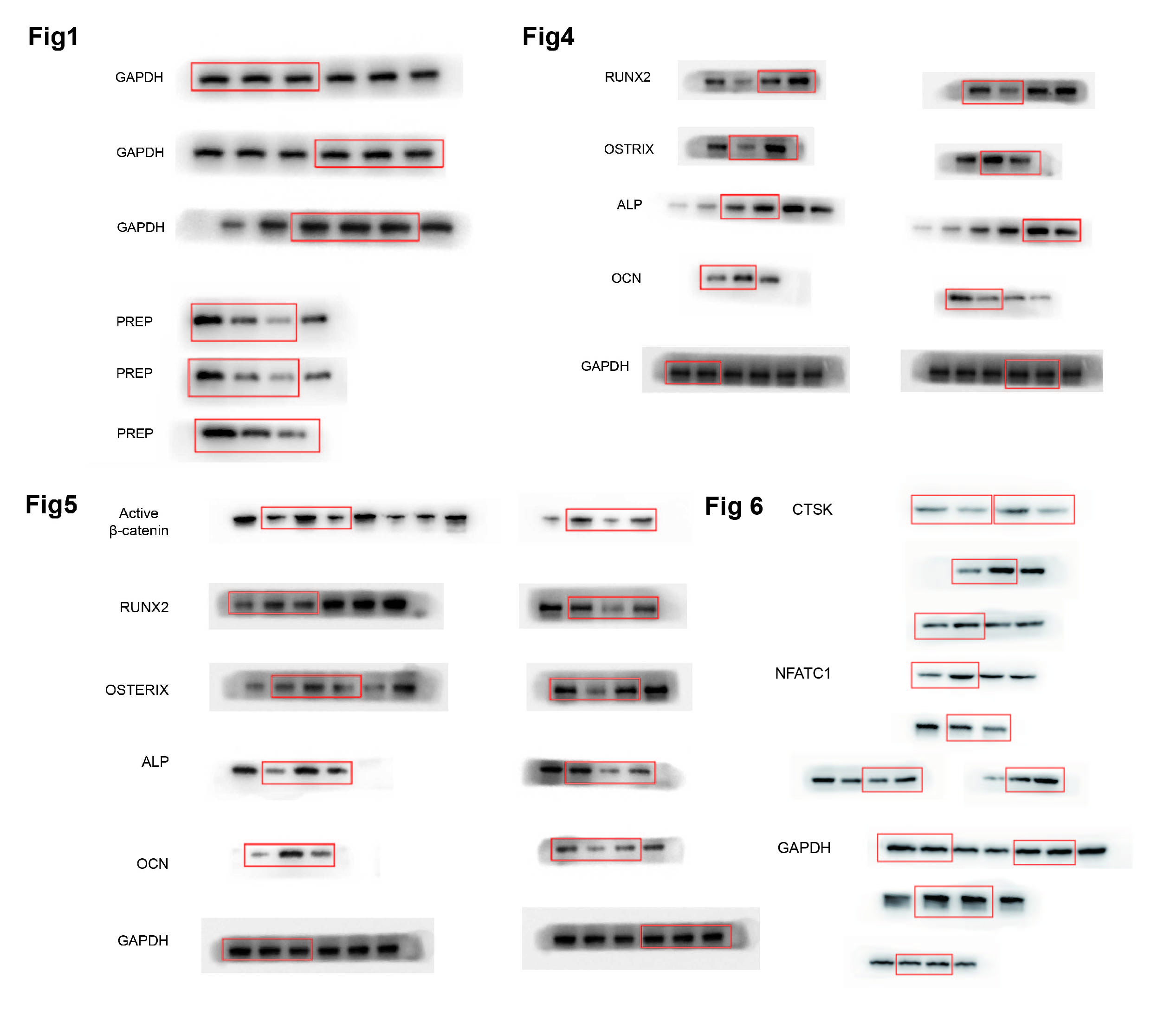

Supplement: Supplementary file 1 — original wb [file 41420_2025_2905_MOESM1_ESM.jpg]
